# Traumatic Encephalopathy Syndrome and Tauopathy in a 19-Year-Old With Child Abuse

**DOI:** 10.1089/neur.2023.0078

**Published:** 2023-12-26

**Authors:** Mike Rueb, Katrin Rauen, Inga Katharina Koerte, Alexandra Gersing, Henrik Zetterberg, Joel Simrén, Matthias Brendel, Kristina Adorjan

**Affiliations:** ^1^ Department of Psychiatry and Psychotherapy, LMU University Hospital, Munich, Germany.; ^2^ Pettenkofer School of Public Health, Munich, Germany.; ^3^ Institute for Medical Information Processing, Biometry and Epidemiology, LMU University Hospital, LMU Munich, Munich, Germany.; ^4^ Center for International Health (CIH LMU), LMU University Hospital, LMU Munich, Munich, Germany.; ^5^ Department of Geriatric Psychiatry, Psychiatric Hospital Zurich, University of Zurich, Zurich, Switzerland.; ^6^ Institute for Stroke and Dementia Research (ISD), LMU University Hospital, LMU Munich, Munich, Germany.; ^7^ Psychiatric Neuroimaging Laboratory, Brigham and Women's Hospital, Massachusetts General Hospital, Harvard Medical School, Boston, Massachusetts, USA.; ^8^ Department of Child and Adolescent Psychiatry, Psychosomatic, and Psychotherapy, LMU University Hospital, Munich, Germany.; ^9^ Department of Psychiatry, Massachusetts General Hospital, Harvard Medical School, Boston, Massachusetts, USA.; ^10^ Department of Neuroradiology, LMU University Hospital, LMU Munich, Munich, Germany.; ^11^ Department of Psychiatry and Neurochemistry, Institute of Neuroscience and Physiology, the Sahlgrenska Academy at the University of Gothenburg, Mölndal, Sweden.; ^12^ Clinical Neurochemistry Laboratory, Sahlgrenska University Hospital, Mölndal, Sweden.; ^13^ Department of Neurodegenerative Disease, Institute of Neurology, University College London, London, United Kingdom.; ^14^ UK Dementia Research Institute, University College London, London, United Kingdom.; ^15^ Hong Kong Center for Neurodegenerative Diseases, Hong Kong, China.; ^16^ Wisconsin Alzheimer's Disease Research Center, University of Wisconsin School of Medicine and Public Health, University of Wisconsin–Madison, Madison, Wisconsin, USA.; ^17^ Department of Nuclear Medicine, LMU University Hospital, LMU Munich, Munich, Germany.; ^18^ German Center for Neurodegenerative Diseases (DZNE) Munich, Germany.; ^19^ Munich Cluster for Systems Neurology (SyNergy), Munich, Germany.; ^20^ Institute of Psychiatric Phenomics and Genomics, LMU University Hospital, Munich, Germany.

**Keywords:** cognitive decline, physical child abuse, PI-2620, tau PET, tau protein, traumatic encephalopathy syndrome

## Abstract

The majority of traumatic encephalopathy syndrome (TES) cases have been reported in former contact sport athletes. This is the first case with TES in a 19-year-old male patient with progressive cognitive decline after daily domestic physical violence through repeated hits to the head for 15 years. The patient presented with a moderate depressive episode and progressive cognitive decline. Tau positron emission tomography (PET) with 220 MBq of [^18^F]PI-2620 revealed increased focal signal at the frontal and parietal white/gray matter border. Brain magnetic resonance imaging (MRI) showed a cavum septum pellucidum, reduced left-sided hippocampal volume, and a left midbrain lesion. Cerebrospinal fluid results showed elevated total and p-tau. Neurocognitive testing at admission showed memory deficits clearly below average, and hampered dysfunctions according to the slow processing speed with a low mistake rate, indicating the acquired, thus secondary, attentional deficits. We diagnosed the patient with a TES suggestive of chronic traumatic encephalopathy and classified him as having subtle/mild functional limitation with a most likely transition to mild dementia within the TES criteria. This report underlines child abuse as a relevant criterion in diagnosing TES in cases with repetitive hits to the head. In addition to clinical markers, we show the relevance of fluid tau biomarkers and tau-PET to support the diagnosis of TES according to the recently published diagnosis criteria for TES.

## Introduction

A causal correlation between child maltreatment and mental disorders is well known.^1^ The long-term effects of repeated hits to the head gain increased attention because of the mounting evidence of tauopathies and neurodegenerative disorders associated with exposure to repeated hits to the head.^2^ Recently, a consensus statement on the diagnostic criteria of traumatic encephalopathy syndrome (TES) was published.^3^ Most reported cases of TES have been found in former contact sport athletes or military veterans.^2^ Chronic traumatic encephalopathy (CTE) has previously been found post-mortem in victims of physical abuse.^4^

Here, we report the first case with TES suggestive of CTE in a 19-year-old male patient after daily domestic physical violence through repeated hits to the head for 15 years until age 16. The patient presented with a depressive syndrome, progressive failure in professional and daily activities over the past 12 months, as well as subjective, progressive cognitive dysfunction since approximately 3 years. At admission, there were severe attentional, concentration (onset at age 7), and memory deficits (onset at age 12), depressed mood, reduced activity, rumination, feelings of worthlessness, negative prospects, and insomnia for 3 years. The patient reported being neglected, emotionally (blaming, ridiculing, rejecting, threatening, and frightening) and physically (repeated hits to the head with her hand, fist, and sometimes with objects such as a wooden washing spoon) abused daily by his mother for 15 years based on self-report as well as next-to-kin reports provided by his father (Supplementary Table S3).

## Methods and Results

**FIG. 1.**
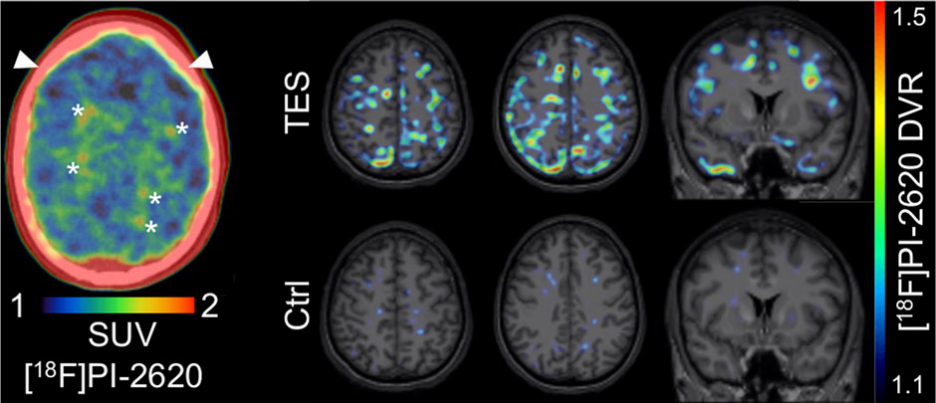
Tau-PET. Left, static tau-PET/CT image (20–40 min p.i.) as assessed in clinical routine, indicating focal lesions of increased tracer uptake at the frontal and parietal white/gray matter border (*) and elevated skull uptake (white arrows). Right, parametric semiquantitative tau-PET images derived from the full 60-min scan (DVR), upon an MRI template (masked extracerebral structures). Upper row shows the patient, lower row shows a tau-negative control (47 years, male). Semiquantitative comparison confirmed elevated focal tau-PET signal predominantly observed at the white/gray matter border in the patient. A dynamic 60-min PET emission was recorded upon bolus (10-sec) tracer injection using a Siemens Biograph 64 PET/CT scanner (Siemens Healthineers, Erlangen, Germany). A low-dose CT served for attenuation correction. Imaging data were reconstructed using an iterative OSEM3D algorithm and binned into 35 frames (12 × 5, 6 × 10s, 3 × 20s, 7 × 60s, 4 × 300s, 3 × 600s). Multi-linear reference tissue modeling 2 was performed using the cerebellum (excluding the dentate nucleus and superior layers) as a reference tissue. For simplified quantification, the 20- to 40-min SUV was calculated. Parametric data of the patient were visually compared to a cognitively unimpaired 47-year-old male after projection upon an MRI template. CT, computed tomography; DVR, distribution volume ratio; MRI, magnetic resonance imaging; PET, positron emission tomography; SUV, standard uptake value.

**FIG. 2.**
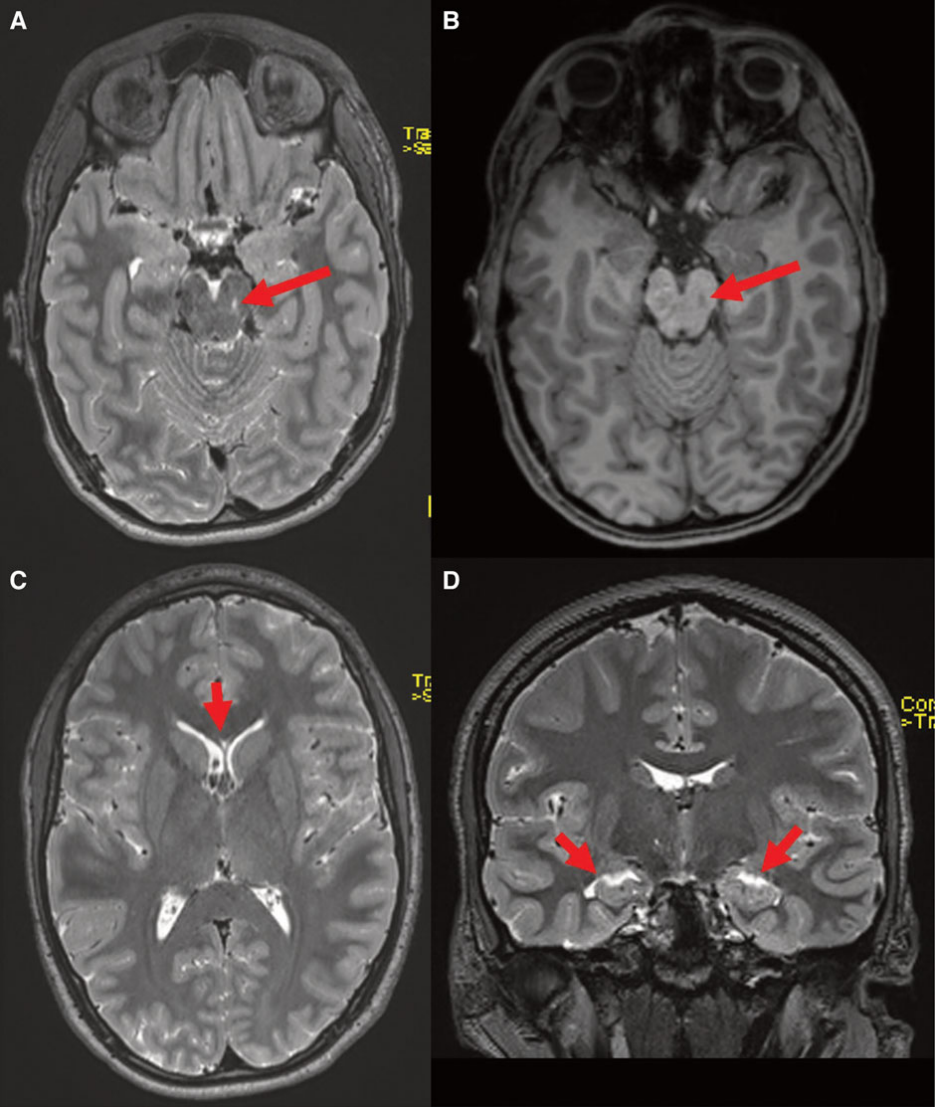
Brain MRI. Brain MRI showing a left midbrain lesion: an axial 2-mm reformation of the 3D T2 sequence (**A**) with a faint crescenteric focus of T2 signal hyperintensity (red oblique arrow). This correlates with the T1 signal hypointensity (red oblique arrow) of the 3D T1 GRE sequence (**B**). Further, an axial 2-mm reformation of the 3D T2 sequence (**C**) revealed the presence of a cavum septum pellucidum (total length, 14 mm; vertical red arrow). The coronal 2-mm reformation of the 3D T2 sequence (**D**) shows subtle widening of the temporal horns of the lateral ventricles bilaterally and a slight reduction in volume of the hippocampi (red oblique arrows). Brain MRI was performed using the following sequences: 3D T1 gradient echo (GRE); 3D diffusion tensor imaging (DTI); 3D T2 turbo spin echo (TSE); 3D fluid-attenuated inversion recovery (FLAIR); coronal T2-weighted (2-mm slice thickness); axial T2*; and SWI. Images were assessed using a structured report based on the NINDS Common Data Elements (CDEs) for traumatic brain injury. Automated volumetric segmentation was performed using the 3D T1 GRE sequence and the software-tool md.brain v1.1.1, which compares individual values to a normative database comprised of several thousand persons 18–92 years of age while accounting for age, sex, and intracranial volume. 3D, three-dimensional; MRI, magnetic resonance imaging; NINDS, National Institute of Neurological Disorders and Stroke; SWI, susceptibility weighted imaging.

We performed a tau-PET (positron emission tomography) with 220 MBq of [^18^F]PI-2620 that showed an increased focal PI-2620 binding at the frontal and parietal white/gray matter border (see Fig. 1). Isolated cortical areas were observed with moderate focally increased binding (e.g., frontal gyrus superior left). Significantly increased binding was observed in the skull, most likely reflecting bone marrow activation. Brain magnetic resonance imaging (MRI) showed a cavum septum pellucidum, reduced left-sided hippocampal volume, and a left midbrain lesion (Fig. 2). Cerebrospinal fluid (CSF) results showed elevated total and p-tau (Supplementary Table S2). Neurocognitive testing at admission showed memory deficits clearly below average, and hampered dysfunctions according to the slow processing speed with a low mistake rate, indicating the acquired, thus secondary, attentional deficits (Supplementary Table S1). Additional clinical details are provided in the Supplementary Appendix.

## Discussion

This case highlights TES as a relevant differential diagnosis in such clinical cases with unclear attention deficit disorder or depression with loss of independence in daily activities and progressive cognitive burdens in the context of child abuse.

It remains to be shown that next-generation tau-PET tracers overcome the limited agreement of [^18^F]flortaucipir binding with post-mortem neuropathological findings in CTE. Importantly, some next-generation tau-PET tracers show a strong affinity to 4-repeat tau, which is predominantly present in mild disease.^5^ Thus, [^18^F]PI-2620^6^ may have more potential to detect tau deposition in mild CTE cases compared to [^18^F]RO-948 and [^18^F]MK-6240, which did not indicate relevant binding in 4-repeat tauopathies *in vivo* or *in vitro*.

With brain MRI, presence of a cavum septum pellucidum was revealed, which has previously been reported in persons at high risk for CTE.^7^ The reported patient demonstrated a significantly reduced volume of both hippocampi in comparison to an age-matched cohort, in synopsis to the otherwise normal parenchymal volume in the other brain regions. This may indicate a subtle, beginning atrophy of the medial temporal lobes, which again is a potential early *in vivo* imaging marker to identify patients at high risk for CTE.^8^

Given that there are no established biomarkers for CTE, the lumbar puncture is not necessary for the diagnosis of TES as per the consensus guideline. However, obtaining values for biomarkers of neurodegeneration (neurofilament light chain; NfL), in both CSF and plasma, allowed us to measure both the presence and have a baseline value to track future development of neuronal injury, given that both the absolute values of CSF and plasma NfL as well as the rate of change in NfL have been found to be increased in neurodegenerative disorders.^9^ In addition, the increased CSF tau concentrations suggest central nervous system tau pathophysiology, which is a defining feature of CTE,^10^ although the link between tau pathology in CTE and CSF tau biomarkers has not yet been established.^10^

## Conclusion

Our findings showed the clinical relevance of the additional diagnostics, including modern tau biomarkers and tau-PET, to investigate the suspicion of TES according to the recently published diagnosis criteria for TES, given that we found several surrogates of tau accumulation in the brain even in this adolescent patient. The value of next-generation tau-PET tracers should be evaluated in cases of suspected TES, given that the combination of neuroimaging, fluid biomarkers, and monitoring of neurobehavioral symptoms may strengthen the diagnosis of TES at an early stage. We encourage clinicians to consider TES as a differential diagnosis when diagnosing a patient with unspecific symptoms and a history of exposure to repeated hits to the head. Further, we suggest expanding the current version of the diagnostic criteria of TES^3^ to include repeated hits to the head in the context of physical abuse.

## Supplementary Material

Supplemental data

Supplemental data

Supplemental data

Supplemental data

## TES Study Group

Jenny Zeumer, Department of Psychiatry and Psychotherapy, LMU University Hospital, Munich, Germany; Nadia Bieler, Department of Psychiatry and Psychotherapy, LMU University Hospital, Munich, Germany; Oemer Faruk Oeztuerk, Department of Psychiatry and Psychotherapy, LMU University Hospital, Munich, Germany, and International Max Planck Research School for Translational Psychiatry, Munich, Germany; Tilman Bondzio, Department of Psychiatry and Psychotherapy, LMU University Hospital, Munich, Germany, and Schoen Clinic Roseneck, Prien am Chiemsee, Germany; Annedore Bodenbender, Department of Psychiatry and Psychotherapy, LMU University Hospital, Munich, Germany; Andreas Behringer, Department of Psychiatry and Psychotherapy, LMU University Hospital, Munich, Germany; Maximilian Scheifele, Department of Nuclear Medicine, LMU University Hospital, Munich, Germany; Gabriela Latour, Department of Geriatric Psychiatry, Psychiatric Hospital Zurich, University of Zurich, Switzerland.

## Abbreviations Used

CSF: cerebrospinal fluid
CTE: chronic traumatic encephalopathy
MRI: magnetic resonance imaging
NfL: neurofilament light chain
PET: positron emission tomography
TES: traumatic encephalopathy syndrome
