# Supplementary material for: Traumatic Encephalopathy Syndrome and Tauopathy in a 19-Year-Old With Child Abuse
Source: Neurotrauma Rep. 2023 Dec 26;4(1):857–62. doi: 10.1089/neur.2023.0078 (PMC10754342; doi:10.1089/neur.2023.0078)
Supplement: Supplemental data [file Suppl_TableS3.docx]

*Table S3: Diagnostic Criteria for TES*

| **NINDS Consensus Diagnostic Criteria on TES** | | | | **Clincial findings of the 19-year-old patient** | |
| --- | --- | --- | --- | --- | --- |
| **Table** | **Title** | **Criteria** | **Sub-criteria** | **Assessment** | **Comment** |
| **Table 1** | **Substantial exposure to repetitive head impact** | sport | | no | no contact sport in history |
|  |  | military | | no | no military service in history |
|  |  | other (domestic violence) | | yes | daily domestic physical violence to the head from age 2 until age of 16, in total 15 years (hits by hand, fist, wooden spoon) |
| **Table 2** | **Core clinical features** | Cognitive impairment | self, informant or clinician's report | yes | self, father, clinicians |
|  |  |  | decline baseline | yes | decline begun during the period of repetitive head impact exposure (around age of 6/7) |
|  |  |  | decline in episodic memory/executive functioning | yes | verbal and short-time memory deficit, clinically episodic memory deficit (biographical landmarks regarding family), executive dysfunction *e.g.* slow processing speed |
|  |  |  | performance on neuropsychological testing | yes | NPT: no episodic deficit, MoCA 27/30, QOLIBRI subscales of cognition (43/100), RPQ (manifest though mild post-concussive syndrome), at least F06.7 mild cognitive impairment; probable F07.2 organic syndrome with cognitive and behavioral changes after head trauma |
|  |  | Neurobehavioural dysregulation | self, informant or clinician's report | yes | self, father, clinicians |
|  |  |  | decline baseline | yes | decline begun during the period of repetitive head impact exposure (around age of 6/7) |
|  |  |  | poor regulation or control of emotions/behaviour | yes | poor regulation of emotions, emotionlessness, emotional numbness, apathy, inhibited drive |
|  |  | Progressive course | | yes | after age 16 decline (according to patient and father), increased stressors (traineeship, lives alone) |
| **Table 3** | **Not fully accounted for by other disorders** | Cognitive deficits | | yes | unlikely ADD by birth/early development, acquired ADD cannot be fully excluded (low error rate, works slowly), methylphenidate did not improve symptoms, atomoxetine just slightly, short-term memory loss |
|  |  | Neurobehavioural dysregulation | | yes | still present after depressive episode has regressed |
|  |  | Comorbid neurodegenerative diagnosis | | yes | other neurodegenerative diseases excluded or unlikely |
|  |  | Comorbid diagnosis of substance use, PTSD, mood or anxiety disorder | | yes | recurrent moderate depressive disorder (remitted after two weeks as an inpatient) |
| **Table 4** | **Level of functional dependance/dementia** | independent | | no |  |
|  |  | subtle/mild functional limitation | | yes | failure in professional education, reduced performance in job, household responsibilities, social roles, partly dependent in instrumental ADLs (managing money, paying bills, completing taxes, cleaning flat, preparing meals) |
|  |  | mild dementia | | (yes) | not fully independent in basic ADLs on admission (personal hygiene, brushing teeth, showering), improved on ward after a month |
|  |  | moderate dementia | | no |  |
|  |  | severe dementia | | no |  |
| **Table 5** | **Supportive features** | Delayed onset | | (yes) | probably onset of hits to the head at age 2/3, delayed onset of symptomatology at age 6/7 |
|  |  | Motor signs | | no |  |
|  |  | Psychiatric features | | yes | no anxiety, apathy, recurrent depression (moderate level on admission) |
| **Table 6** | **Provisional Levels of certainty for CTE pathology** | Suggestive of CTE | | yes | meets criteria of 'probable' but no contact sport in history, therefore suggestive according to flow diagram |

*Legend: According to the National Institute of Neurological Disorders and Stroke (NINDS) Consensus Diagnostic Criteria for Traumatic Encephalopathy Syndrome*^28^*. The criteria were assessed independently by three clinicians of the team (MR, KR, KA) and discussed with IK. Yes: meaning the criterion is fulfilled; (yes): the criterion is likely to be fulfilled; and no: the criterion is not fulfilled*
