# Supplementary material for: Traumatic Encephalopathy Syndrome and Tauopathy in a 19-Year-Old With Child Abuse
Source: Neurotrauma Rep. 2023 Dec 26;4(1):857–62. doi: 10.1089/neur.2023.0078 (PMC10754342; doi:10.1089/neur.2023.0078)
Supplement: Supplemental data [file Suppl_TableS2.docx]

Table S2: Biomarkers in CSF and blood plasma

| **Biomarker** | **[pg/mL]** | **Reference limit** |
| --- | --- | --- |
| CSF Aβ40 | 17607 | - |
| CSF Aβ42 | 1817 | > 620 pg/mL (> 50 years) |
| CSF (Aβ42/Aβ40)*10 | 1.04 | > 0.72 (> 50 years) |
| CSF total tau | 418 | < 409 pg/mL (> 50 years) |
| CSF phosphorylated tau 181 | 71.5 | < 50 pg/mL (> 50 years) |
| CSF neurofilament light | 186 | < 380 pg/mL |
| CSF glial fibrillary acidic protein | 250 | < 750 pg/mL |
| Plasma neurofilament light | 3.62 | < 20 pg/mL |

Legend: Biomarkers in CSF (cerebrospinal fluid) and blood plasma of the patient from March 2021. CSF Aβ40, Aβ42, total tau and phosphorylated tau (amino acid 181) concentrations were measured using Lumipulse^16^. CSF neurofilament light and glial fibrillary acidic protein concentrations were measured using in-house enzyme-linked immunosorbent assays^30^. Plasma neurofilament light concentration was measured using the commercially available NF-Light assay on a Single molecule array (Simoa) HD-X Analyzer (Quanterix, Billerica, MA)
