# Supplementary material for: Traumatic Encephalopathy Syndrome and Tauopathy in a 19-Year-Old With Child Abuse
Source: Neurotrauma Rep. 2023 Dec 26;4(1):857–62. doi: 10.1089/neur.2023.0078 (PMC10754342; doi:10.1089/neur.2023.0078)
Supplement: Supplemental data [file Suppl_TableS1.docx]

Table S1: Psychometric assessments at admission and discharge

|  | **Subscales** | **Rating** | **Reference** | **at admission** | **at discharge**^‡^ |
| --- | --- | --- | --- | --- | --- |
| BDI | | S^*^ | 0-12 no/13-19 mild depression | 15/63 | 2/63 |
| HAM-D | | E^†^ | 0-84/≤ 7 not depressed | 17/84 | 1/84 |
| HAM-A | | E | 0-56/< 17 mild severity | 5/56 | 6/56 |
| QOLIBRI | *total* | S | 0-100/≥ 60 satisfied HRQoL | 68/100 | 70/100 |
|  | *cognition* | S | 0-100/≥ 60 satisfied HRQoL | 43/100 | - |
|  | *self* | S | 0-100/≥ 60 satisfied HRQoL | 54/100 | 75/100 |
|  | *daily life & autonomy* | S | 0-100/≥ 60 satisfied HRQoL | 50/100 | 64/100 |
| PSQI |  | S | 0-21/≤ 5 good sleep quality | 15/21 | 7/21 |
| RPQ |  | S | manifest though mild post-concussive syndrome | 16/64 | 18/64 |
| MoCA |  | E | 0-30/normal ≥ 26 | 27/30 | 29/30 |

Footnotes: *S (self-rating), †E (external rating); ‡ (after three months of in-patient treatment), BDI (Beck’s Depression Inventar); HAM-D (Hamilton Rating Scale for Depression); HAM-A (Hamilton Anxiety Rating Scale); QOLIBRI (Quality of Life after Brain Injury); PSQI (Pittsburgh Sleep Quality Index); RPQ (Rivermead post-concussion symptoms questionnaire); MoCA (Montreal Cognitive Assessment)
