# Supplementary material for: Traumatic Encephalopathy Syndrome and Tauopathy in a 19-Year-Old With Child Abuse
Source: Neurotrauma Rep. 2023 Dec 26;4(1):857–62. doi: 10.1089/neur.2023.0078 (PMC10754342; doi:10.1089/neur.2023.0078)
Supplement: Supplemental data [file Suppl_Appendix.docx]

**Supplementary appendix**

[Patient information 2](#_Toc143091687)

[Patient history and clinical findings 2](#_Toc143091688)

[Medical, family, and psychosocial history and past interventions with outcomes and timeline 2](#_Toc143091689)

[Pregnancy and child development 2](#_Toc143091690)

[Neglect 2](#_Toc143091691)

[Abuse 2](#_Toc143091692)

[Psychiatric history 3](#_Toc143091693)

[Past interventions 3](#_Toc143091694)

[Sports 3](#_Toc143091695)

[Diagnostic Assessment 4](#_Toc143091696)

[Psychometric assessment 4](#_Toc143091697)

[Neurocognitive testing 4](#_Toc143091698)

[Occupational therapy assessment 5](#_Toc143091699)

[Molecular imaging 5](#_Toc143091700)

[Magnetic resonance imaging 6](#_Toc143091701)

[Biomarkers 6](#_Toc143091702)

[Diagnosis 7](#_Toc143091703)

[Prognosis 8](#_Toc143091704)

[Tables 8](#_Toc143091705)

[List of abbreviations 17](#_Toc143091706)

[References 17](#_Toc143091707)

# Patient information

## Patient history and clinical findings

A 19-year-old male presented with a depressive syndrome, progressive failure in professional and daily activities over the past twelve months. At admission, he presented with severe symptoms of attention, concentration (known since the age of seven) and memory deficits (since about the age of twelve), depressed mood, reduced activity, rumination, feelings of worthlessness, negative future prospects, insomnia for two to three years (difficulty falling asleep for 30-60 min) and sleep disturbances (three to five times a night). The patient was admitted for reevaluation of his pharmacological treatment (methylphenidate hydrochloride), as well as for further diagnostics and therapy.

## Medical, family, and psychosocial history and past interventions with outcomes and timeline

### Pregnancy and child development

The parents separated when he was three, after that the patient lived with his mother until the age of 16.

### Neglect

The patient reported being neglected and emotionally abused (blaming, ridiculing, rejecting, threatening, frightening) by his mother.

### Abuse

According to the patient and his father, from the age two until 16, the patient was hit daily on the head by the mother with her hand, fist, and sometimes with objects such as a wooden washing spoon, so that he sometimes hit his head on the floor or on furniture. At the age of three, the mother threw him through the kitchen onto the floor and against a piece of furniture (unclear how often, at least two to three times, according to the father). Between the age of three and five, the mother left him alone in the apartment for several nights. The patient did not talk about the abuse until the age of 16.

### Psychiatric history

At age seven, he was diagnosed with simple disorder of activity and attention (ADD) (ICD-10-GM: F90.0G, DSM-5/ICD-10-CM: F90.0). At age 16, he was diagnosed with reduced concentration (ICD-10-GM: F98.8G, DSM-5/ICD-10-CM: F98.8) and attention deficit hyperactivity disorder (ICD-10-GM: F90.0G, DSM-5/ICD-10-CM: F90.0).

### Past interventions

At age nine, he was given methylphenidate hydrochloride (Medikinet^®^ retard) 20 mg per day.

Due to the disclosure of the abuse and progression of symptoms brain magnetic resonance imaging (MRI) without contrast was performed (T2 weighted, T1 weighted, susceptibility weighted imaging (SWI)) at age 16. The brain MRI did not show any evidence of contusion, microhemorrhage, or focal lesion. Consequently, they increased the methylphenidate hydrochloride to 25 and 30 mg.

At age 18, he discontinued methylphenidate hydrochloride 30 mg by choice due to adverse drug effects including loss of appetite and depressed mood. Therefore, and because his profession was affected by the SARS-CoV-2 pandemic, he dropped out of his traineeship about six months later. One year after stopping the medication, he presented himself at the LMU university psychiatric clinic for a pharmacological reevaluation. Since then, his attention, concentration and memory deficit worsened.

### Sports

He had no history of participating in contact sports.

# Diagnostic Assessment

## Psychometric assessment

Beck’s Depression Inventory (BDI) and Hamilton Rating Scale for Depression (HAM-D) showed a major depression on admission and remission of symptoms before discharge.

Assessing the health- and disease specific quality of life (HRQoL) by using the Quality of life after brain injury (QOLIBRI^1^) instrument showed a total score of 68 out of 100, indicating a satisfied overall HRQoL. However, subscales of *cognition* (43/100), *self* (54/100), and *daily life & autonomy* (50/100) pinpointed unsatisfied HRQoL, whereas subscales of *social relationships* (67/100), *emotions* (80/100), and *physical problems* (90/100) underline good HRQoL. In particular, the latter two subscales represent limitations from which the patient did not suffer. Before discharge, the subscales of *self* (75/100) and *daily life & autonomy* (64/100) improved.

Poor sleep quality indicated by self-assessment using the Pittsburgh sleep quality index (PSQI)^2^. The patient suffered from poor sleep quality for two to three years and 90 days after admission although he had been taking medication (15 mg of mirtazapine, 25 mg of agomelatine), indicating the chronicity and severity of burden or the major depression which was not sufficiently treated.

Rivermead post-concussion symptoms questionnaire (RPQ)^3^ showed a manifest though mild post-concussive syndrome.

We considered the score of 27/30 in the MoCA for a 19-year-old to be conspicuous in conjunction with HRQoL subscale of cognition. See Table S1.

## Neurocognitive testing

The neurocognitive testing at admission showed memory deficits clearly below average in the Auditory Verbal Learning Test (AVLT)^4^, and hampered dysfunctions according to the slow processing speed with low mistake rate, indicating the acquired, thus secondary attentional deficits. The Repeatable Battery for the Assessment of Neuropsychological Status (RBANS)^5^, for recording various cognitive sub-performances, revealed an average verbal-serial learning performance for a word list. Active recall was below average. When learning and later recalling a short story, the performance was below average in each case, whereby the memory performance was only just below the norm. The visuomotor speed, measured with the ‘symbol-number test’, was at the lower end of the normal range. Word fluency was low. In the Trail Making Test B (TMT-B)^6^, which measures cognitive flexibility, his performance was in the low range.

## Occupational therapy assessment

The cognitive deficits were less significant in practical activities. The greatest limitations became apparent in retentiveness and memory performance, which the patient also noticed himself. The limitations in memory performance led to difficulties in structuring everyday life and adequate time planning.

## Molecular imaging

We performed a tau-PET with 220 MBq [^18^F]PI-2620^7^ that showed an increased focal PI-2620 binding at the frontal and parietal white-grey matter border (see Figure 1). Isolated cortical areas with moderate focally increased binding, *e.g.*, frontal gyrus superior left. Significantly increased binding was observed in the skull, most likely reflecting bone marrow activation.

Some next generation tau-PET tracers show a strong distinct affinity to 4-repeat tau which is predominantly present in mild disease^8^, indicating in vitro binding and positive in vivo signal for [^18^F]PM-PBB3^9^ and [^18^F]PI-2620^7,10^ whereas [^18^F]RO-948^11^ and [^18^F]MK-6240^12^ did not indicate relevant in vivo binding in 4-repeat tauopathies or in vitro^13^. Tau-PET with [^18^F]PI-2620 has not yet been validated to substantiate clinical diagnosis of TES, so imaging should be acknowledged as a surrogate of tau accumulation together with elevated levels of p-tau in CSF.

## Magnetic resonance imaging

The brain MRI showed a slightly asymmetric ventricular system (see Figure 2). Presence of a cavum septum pellucidum was noted (length: 14mm). On SWI and T2*-weighted images, no microhemorrhages or superficial siderosis detected. One T2-hyperintense lesion in the left midbrain. Regular stratification and grey-white differentiation of the hippocampi. The volumetric analysis revealed a reduction in volume by more than two standard deviations of the left hippocampus (percentile: 0.5% (4.0ml; normal age- and sex-adjusted range: 4.1-5.5ml). The right hippocampus revealed a decreased volume of the hippocampus, which was considered to be within two standard deviations (4.2%; 4.2ml (normal age- and sex-adjusted range: 4.1ml-5.4ml)). The overall brain volume was regarded as within the normal range (overall percentile: 95.1%, 1470.4ml (normal age- and sex-adjusted range: 1337.6-1482.8ml); overall white matter percentile: 91.7%, 598.6ml (normal age- and sex-adjusted range: 513.2-614.1ml); overall grey matter percentile: 83.2%, 871.8ml (normal age- and sex-adjusted range: 784.2-902.4ml)). There was no change of the morphological findings noted between the three available MRIs for three months. Automated volumetric segmentation was performed using the 3D T1 GRE sequence and the software-tool md.brain v1.1.1 which compares individual values to a normative database comprised of several thousand individuals aged 18-92 years while accounting for age, sex, and intracranial volume.^14,15^ Due to the very subtle findings the brain volume of the different regions was assessed for verification.

## Biomarkers

Total tau was increased in the cerebrospinal fluid, as measured by Lumipulse enzyme-linked immunosorbent assay^16^. Phosphorylated tau 181 (p-tau)^16^, was also slightly above the cut-off for age 50 and older (see Table S2). Since p-tau181 is not as widely used in younger individuals, no validated age-related reference limits exist for this age-category. Based on earlier studies, however, CSF p-tau181 concentrations are lower in young adults as compared with healthy elderly^17,18^. Thus, it is reasonable to assess the concentration of CSF p-tau181 measured as abnormal as compared to healthy individuals of comparable age^18^.

As there are no established biomarkers for CTE^19,20^, it is debatable to what extent a lumbar puncture was necessary. However, obtaining values for biomarkers of neurodegeneration (NfL) in both CSF and plasma, allowed us to measure both the presence and have a baseline value to track future development of neuronal injury, since both the absolute values of CSF^21^ and plasma NfL^22^ as well as the rate of change in NfL^23^ have been found to be increased in neurodegenerative disorders. P- and t-tau are widely accepted analyses that reflect abnormal tau metabolism of in Alzheimer’s disease. However, it is not yet known how well these biomarkers reflect the neuropathology of CTE^24,25^. Due to the non-existence of reference values for young age, the informative value of the biomarkers is limited. However, in recent studies performed in well-characterized cohorts, the concentrations of CSF p-tau181 and t-tau are lower in young adults (⁓20 years of age) as compared to healthy elderly, likely reflecting normal age-related increases^17^. Consequently, a clinical cut-off in young adults would likely be lower than the one presented here, which is based on a material of older adults^26^. A final diagnosis can only be made neuropathologically post-mortem^27^.

# Diagnosis

According to the National Institute of Neurological Disorders and Stroke Consensus Diagnostic Criteria for Traumatic Encephalopathy Syndrome we diagnosed the patient as presenting TES suggestive of CTE^28^ after daily child abuse for 15 years (see Table S3). Based on the ‘Provisional Levels of Certainty for CTE Pathology’^28^ the patient is classified as ‘suggestive of CTE’ due to no contact sports in history. If repeated head impacts due to physical abuse were part of the framework, we would classify the case as ‘probable of CTE’^28^. We classified the patient as having subtle/mild functional limitation with most likely transition to mild dementia within the TES criteria. As the basic activities of daily living (ADL) improved after one month on the ward, they cannot be clearly attributed to TES, but rather to the moderate depressive episode that had been remitted by then. We consider the TES criterion of delayed onset to be fulfilled, but in retrospect we cannot exclude that some of the symptoms were already present in a mild form in earlier years. Even if we assigned the attention and concentration deficit to the cognitive impairments of the TES diagnosis, it cannot be completely ruled out that part of the symptomatology could be assigned to an independent ADD. Therapeutically, this would have no consequence, as we would have and have treated the patient with atomoxetine according to the state of the literature^29^. Since the patient reported little emotional suffering from the abuse or diagnosis, it would be necessary to find out to what extent this is part of his resilience or his lack of emotion and apathy.

In addition, elevated CSF total- and p-tau as well as increased binding of tau-specific tracer in the PET are suggestive of TES.

In addition, we diagnosed a moderate depressive episode (ICD-10-GM: F32.1, DSM-5/ICD-10-CM: F32.1) on admission.

# Prognosis

Since this is an initial description, accurate prognosis is difficult. The fact that the patient had already shown progression since the age of 16 suggests that further progression – even decades later – may occur^29^.

# Tables

Table S1: Psychometric assessments at admission and discharge

|  | **Subscales** | **Rating** | **Reference** | **at admission** | **at discharge**^‡^ |
| --- | --- | --- | --- | --- | --- |
| BDI | | S^*^ | 0-12 no/13-19 mild depression | 15/63 | 2/63 |
| HAM-D | | E^†^ | 0-84/≤ 7 not depressed | 17/84 | 1/84 |
| HAM-A | | E | 0-56/< 17 mild severity | 5/56 | 6/56 |
| QOLIBRI | *total* | S | 0-100/≥ 60 satisfied HRQoL | 68/100 | 70/100 |
|  | *cognition* | S | 0-100/≥ 60 satisfied HRQoL | 43/100 | - |
|  | *self* | S | 0-100/≥ 60 satisfied HRQoL | 54/100 | 75/100 |
|  | *daily life & autonomy* | S | 0-100/≥ 60 satisfied HRQoL | 50/100 | 64/100 |
| PSQI |  | S | 0-21/≤ 5 good sleep quality | 15/21 | 7/21 |
| RPQ |  | S | manifest though mild post-concussive syndrome | 16/64 | 18/64 |
| MoCA |  | E | 0-30/normal ≥ 26 | 27/30 | 29/30 |

Footnotes: *S (self-rating), †E (external rating); ‡ (after three months of in-patient treatment), BDI (Beck’s Depression Inventar); HAM-D (Hamilton Rating Scale for Depression); HAM-A (Hamilton Anxiety Rating Scale); QOLIBRI (Quality of Life after Brain Injury); PSQI (Pittsburgh Sleep Quality Index); RPQ (Rivermead post-concussion symptoms questionnaire); MoCA (Montreal Cognitive Assessment)

Table S2: Biomarkers in CSF and blood plasma

| **Biomarker** | **[pg/mL]** | **Reference limit** |
| --- | --- | --- |
| CSF Aβ40 | 17607 | - |
| CSF Aβ42 | 1817 | > 620 pg/mL (> 50 years) |
| CSF (Aβ42/Aβ40)*10 | 1.04 | > 0.72 (> 50 years) |
| CSF total tau | 418 | < 409 pg/mL (> 50 years) |
| CSF phosphorylated tau 181 | 71.5 | < 50 pg/mL (> 50 years) |
| CSF neurofilament light | 186 | < 380 pg/mL |
| CSF glial fibrillary acidic protein | 250 | < 750 pg/mL |
| Plasma neurofilament light | 3.62 | < 20 pg/mL |

Legend: Biomarkers in CSF (cerebrospinal fluid) and blood plasma of the patient from March 2021. CSF Aβ40, Aβ42, total tau and phosphorylated tau (amino acid 181) concentrations were measured using Lumipulse^16^. CSF neurofilament light and glial fibrillary acidic protein concentrations were measured using in-house enzyme-linked immunosorbent assays^30^. Plasma neurofilament light concentration was measured using the commercially available NF-Light assay on a Single molecule array (Simoa) HD-X Analyzer (Quanterix, Billerica, MA)

*Table S3: Diagnostic Criteria for TES*

| **NINDS Consensus Diagnostic Criteria on TES** | | | | **Clincial findings of the 19-year-old patient** | |
| --- | --- | --- | --- | --- | --- |
| **Table** | **Title** | **Criteria** | **Sub-criteria** | **Assessment** | **Comment** |
| **Table 1** | **Substantial exposure to repetitive head impact** | sport | | no | no contact sport in history |
|  |  | military | | no | no military service in history |
|  |  | other (domestic violence) | | yes | daily domestic physical violence to the head from age 2 until age of 16, in total 15 years (hits by hand, fist, wooden spoon) |
| **Table 2** | **Core clinical features** | Cognitive impairment | self, informant or clinician's report | yes | self, father, clinicians |
|  |  |  | decline baseline | yes | decline begun during the period of repetitive head impact exposure (around age of 6/7) |
|  |  |  | decline in episodic memory/executive functioning | yes | verbal and short-time memory deficit, clinically episodic memory deficit (biographical landmarks regarding family), executive dysfunction *e.g.* slow processing speed |
|  |  |  | performance on neuropsychological testing | yes | NPT: no episodic deficit, MoCA 27/30, QOLIBRI subscales of cognition (43/100), RPQ (manifest though mild post-concussive syndrome), at least F06.7 mild cognitive impairment; probable F07.2 organic syndrome with cognitive and behavioral changes after head trauma |
|  |  | Neurobehavioural dysregulation | self, informant or clinician's report | yes | self, father, clinicians |
|  |  |  | decline baseline | yes | decline begun during the period of repetitive head impact exposure (around age of 6/7) |
|  |  |  | poor regulation or control of emotions/behaviour | yes | poor regulation of emotions, emotionlessness, emotional numbness, apathy, inhibited drive |
|  |  | Progressive course | | yes | after age 16 decline (according to patient and father), increased stressors (traineeship, lives alone) |
| **Table 3** | **Not fully accounted for by other disorders** | Cognitive deficits | | yes | unlikely ADD by birth/early development, acquired ADD cannot be fully excluded (low error rate, works slowly), methylphenidate did not improve symptoms, atomoxetine just slightly, short-term memory loss |
|  |  | Neurobehavioural dysregulation | | yes | still present after depressive episode has regressed |
|  |  | Comorbid neurodegenerative diagnosis | | yes | other neurodegenerative diseases excluded or unlikely |
|  |  | Comorbid diagnosis of substance use, PTSD, mood or anxiety disorder | | yes | recurrent moderate depressive disorder (remitted after two weeks as an inpatient) |
| **Table 4** | **Level of functional dependance/dementia** | independent | | no |  |
|  |  | subtle/mild functional limitation | | yes | failure in professional education, reduced performance in job, household responsibilities, social roles, partly dependent in instrumental ADLs (managing money, paying bills, completing taxes, cleaning flat, preparing meals) |
|  |  | mild dementia | | (yes) | not fully independent in basic ADLs on admission (personal hygiene, brushing teeth, showering), improved on ward after a month |
|  |  | moderate dementia | | no |  |
|  |  | severe dementia | | no |  |
| **Table 5** | **Supportive features** | Delayed onset | | (yes) | probably onset of hits to the head at age 2/3, delayed onset of symptomatology at age 6/7 |
|  |  | Motor signs | | no |  |
|  |  | Psychiatric features | | yes | no anxiety, apathy, recurrent depression (moderate level on admission) |
| **Table 6** | **Provisional Levels of certainty for CTE pathology** | Suggestive of CTE | | yes | meets criteria of 'probable' but no contact sport in history, therefore suggestive according to flow diagram |

*Legend: According to the National Institute of Neurological Disorders and Stroke (NINDS) Consensus Diagnostic Criteria for Traumatic Encephalopathy Syndrome*^28^*. The criteria were assessed independently by three clinicians of the team (MR, KR, KA) and discussed with IK. Yes: meaning the criterion is fulfilled; (yes): the criterion is likely to be fulfilled; and no: the criterion is not fulfilled*

# List of abbreviations

ADD: attention deficit disorder, ADL: activities of daily living, AVLT: Auditory Verbal Learning Test, BDI: Becks Depression Inventar, CDEs: common data elements, CSF: cerebrospinal fluid, CTE: chronic traumatic encephalopathy, DSM-5: Diagnostic and Statistical Manual of Mental Disorders (5^th^ edition), DTI: Diffusion Tensor Imaging, FLAIR: fluid attenuated inversion recovery, GRE: gradient echo, HAM-A: Hamilton Anxiety Rating Scale, HAM-D: Hamilton depression scale, ICD-10: International Classification of Diseases (10^th^ revision), MRI: magnetic resonance imaging, MoCA: Montreal Cognitive Assessment, NfL: biomarkers of neurodegeneration, NINDS: National Institute of Neurological Disorders and Stroke, PET: Positron Emission Tomography, PSQI: Pittsburgh Sleep Quality Index, QOLIBRI: Quality of Life after Brain Injury, RBANS: Repeatable Battery for the Assessment of Neuropsychological Status, RPQ: Rivermead post-concussion symptoms questionnaire, SWI: susceptibility weighted imaging, TES: traumatic encephalopathy syndrome, TMT-B: Trail Making Test B, TSE: turbo spin echo

# References

1. Rauen K, Reichelt L, Probst P, et al. Quality of life up to 10 years after traumatic brain injury: a cross-sectional analysis. *Health Qual Life Outcomes*. 2020;18(1):166. doi:10.1186/s12955-020-01391-3

2. Buysse DJ, Reynolds CF, Monk TH, Berman SR, Kupfer DJ. The Pittsburgh Sleep Quality Index: a new instrument for psychiatric practice and research. *Psychiatry Res*. 1989;28(2):193-213. doi:10.1016/0165-1781(89)90047-4

3. King NS, Crawford S, Wenden FJ, Moss NE, Wade DT. The Rivermead Post Concussion Symptoms Questionnaire: a measure of symptoms commonly experienced after head injury and its reliability. *J Neurol*. 1995;242(9):587-592. doi:10.1007/BF00868811

4. Helmstaedter C, Durwen HF. VLMT: Verbaler Lern- und Merkfähigkeitstest: Ein praktikables und differenziertes Instrumentarium zur Prüfung der verbalen Gedächtnisleistungen. [VLMT: A useful tool to assess and differentiate verbal memory performance.]. *Schweiz Arch Für Neurol Neurochir Psychiatr*. 1990;141(1):21-30.

5. Randolph C, Tierney MC, Mohr E, Chase TN. The Repeatable Battery for the Assessment of Neuropsychological Status (RBANS): Preliminary Clinical Validity. *J Clin Exp Neuropsychol*. 1998;20(3):310-319. doi:10.1076/jcen.20.3.310.823

6. Reitan RM. Validity of the Trail Making Test as an Indicator of Organic Brain Damage. *Percept Mot Skills*. 1958;8(3):271-276. doi:10.2466/pms.1958.8.3.271

7. Brendel M, Barthel H, van Eimeren T, et al. Assessment of 18F-PI-2620 as a Biomarker in Progressive Supranuclear Palsy. *JAMA Neurol*. 2020;77(11):1-13. doi:10.1001/jamaneurol.2020.2526

8. Cherry JD, Esnault CD, Baucom ZH, et al. Tau isoforms are differentially expressed across the hippocampus in chronic traumatic encephalopathy and Alzheimer’s disease. *Acta Neuropathol Commun*. 2021;9(1):86. doi:10.1186/s40478-021-01189-4

9. Tagai K, Ono M, Kubota M, et al. High-Contrast In Vivo Imaging of Tau Pathologies in Alzheimer’s and Non-Alzheimer’s Disease Tauopathies. *Neuron*. 2021;109(1):42-58.e8. doi:10.1016/j.neuron.2020.09.042

10. Palleis C, Brendel M, Finze A, et al. Cortical [18 F]PI-2620 Binding Differentiates Corticobasal Syndrome Subtypes. *Mov Disord Off J Mov Disord Soc*. Published online May 5, 2021. doi:10.1002/mds.28624

11. Leuzy A, Smith R, Ossenkoppele R, et al. Diagnostic Performance of RO948 F 18 Tau Positron Emission Tomography in the Differentiation of Alzheimer Disease From Other Neurodegenerative Disorders. *JAMA Neurol*. 2020;77(8):955-965. doi:10.1001/jamaneurol.2020.0989

12. Aguero C, Dhaynaut M, Normandin MD, et al. Autoradiography validation of novel tau PET tracer [F-18]-MK-6240 on human postmortem brain tissue. *Acta Neuropathol Commun*. 2019;7(1):37. doi:10.1186/s40478-019-0686-6

13. Mueller A, Kroth H, Oden F, et al. Preclinical comparison of the first generation Tau PET tracer AV1451 and two next-generation Tau PET tracers, MK-6240 and PI-2620. In: *European Journal of Nuclear Medicine and Molecular Imaging*. Vol 46. Springer One New York Plaza, Suite 4600, New York, NY, United States; 2019:S229-S230.

14. Dieckmeyer M, Roy AG, Senapati J, et al. Effect of MRI acquisition acceleration via compressed sensing and parallel imaging on brain volumetry. *Magn Reson Mater Phys Biol Med*. 2021;34(4):487-497. doi:10.1007/s10334-020-00906-9

15. Hitziger S, Ling WX, Fritz T, D’Albis T, Lemke A, Grilo J. Triplanar U-Net with lesion-wise voting for the segmentation of new lesions on longitudinal MRI studies. *Front Neurosci*. 2022;16. doi:10.3389/fnins.2022.964250

16. Keshavan A, Wellington H, Chen Z, et al. Concordance of CSF measures of Alzheimer’s pathology with amyloid PET status in a preclinical cohort: A comparison of Lumipulse and established immunoassays. *Alzheimers Dement Amst Neth*. 2021;13(1):e12131. doi:10.1002/dad2.12131

17. Karikari TK, Pascoal TA, Ashton NJ, et al. Blood phosphorylated tau 181 as a biomarker for Alzheimer’s disease: a diagnostic performance and prediction modelling study using data from four prospective cohorts. *Lancet Neurol*. 2020;19(5):422-433. doi:10.1016/S1474-4422(20)30071-5

18. Sjögren M, Vanderstichele H, Agren H, et al. Tau and Abeta42 in cerebrospinal fluid from healthy adults 21-93 years of age: establishment of reference values. *Clin Chem*. 2001;47(10):1776-1781.

19. Hazrati LN, Schwab N. Embracing the Unknown in the Diagnosis of Traumatic Encephalopathy Syndrome. *Neurology*. 2021;96(18):835-836. doi:10.1212/WNL.0000000000011847

20. Asken BM, Tanner JA, VandeVrede L, et al. Multi-Modal Biomarkers of Repetitive Head Impacts and Traumatic Encephalopathy Syndrome: A Clinicopathological Case Series. *J Neurotrauma*. Published online April 28, 2022. doi:10.1089/neu.2022.0060

21. Bridel C, van Wieringen WN, Zetterberg H, et al. Diagnostic Value of Cerebrospinal Fluid Neurofilament Light Protein in Neurology: A Systematic Review and Meta-analysis. *JAMA Neurol*. 2019;76(9):1035-1048. doi:10.1001/jamaneurol.2019.1534

22. Ashton NJ, Janelidze S, Al Khleifat A, et al. A multicentre validation study of the diagnostic value of plasma neurofilament light. *Nat Commun*. 2021;12(1):3400. doi:10.1038/s41467-021-23620-z

23. Preische O, Schultz SA, Apel A, et al. Serum neurofilament dynamics predicts neurodegeneration and clinical progression in presymptomatic Alzheimer’s disease. *Nat Med*. 2019;25(2):277-283. doi:10.1038/s41591-018-0304-3

24. Zetterberg H, Blennow K. Fluid biomarkers for mild traumatic brain injury and related conditions. *Nat Rev Neurol*. 2016;12(10):563-574. doi:10.1038/nrneurol.2016.127

25. Asken BM, Tanner JA, VandeVrede L, et al. Plasma P-tau181 and P-tau217 in Patients With Traumatic Encephalopathy Syndrome With and Without Evidence of Alzheimer Disease Pathology. *Neurology*. Published online May 16, 2022:10.1212/WNL.0000000000200678. doi:10.1212/WNL.0000000000200678

26. Gobom J, Parnetti L, Rosa-Neto P, et al. Validation of the LUMIPULSE automated immunoassay for the measurement of core AD biomarkers in cerebrospinal fluid. *Clin Chem Lab Med*. 2022;60(2):207-219. doi:10.1515/cclm-2021-0651

27. McKee AC, Cairns NJ, Dickson DW, et al. The first NINDS/NIBIB consensus meeting to define neuropathological criteria for the diagnosis of chronic traumatic encephalopathy. *Acta Neuropathol (Berl)*. 2016;131(1):75-86. doi:10.1007/s00401-015-1515-z

28. Katz DI, Bernick C, Dodick DW, et al. National Institute of Neurological Disorders and Stroke Consensus Diagnostic Criteria for Traumatic Encephalopathy Syndrome. *Neurology*. Published online March 15, 2021. doi:10.1212/WNL.0000000000011850

29. Cantu R, Budson A. Management of chronic traumatic encephalopathy. *Expert Rev Neurother*. 2019;19(10):1015-1023. doi:10.1080/14737175.2019.1633916

30. Gaetani L, Höglund K, Parnetti L, et al. A new enzyme-linked immunosorbent assay for neurofilament light in cerebrospinal fluid: analytical validation and clinical evaluation. *Alzheimers Res Ther*. 2018;10(1):8. doi:10.1186/s13195-018-0339-1
